# Maternal Smoking Induced Cardiovascular Risks in Fetuses: How Can *in silico* Models Help?

**DOI:** 10.3389/fbioe.2020.00097

**Published:** 2020-02-19

**Authors:** Harvey Ho, Hongchao Guo, Shawn Means, Jing Tang, Peter Hunter

**Affiliations:** ^1^Auckland Bioengineering Institute, The University of Auckland, Auckland, New Zealand; ^2^Stanford Cardiovascular Institute, Stanford University, Stanford, CA, United States; ^3^School of Natural and Computational Sciences, Massey University, Auckland, New Zealand; ^4^Chongqing Health Center for Women and Children, Chongqing, China

**Keywords:** maternal smoking, nicotine, *in silico* model, umbilical artery, fetus

## Introduction

Despite mounting evidence of detrimental effects of maternal smoking on fetuses, including still birth, intrauterine growth restriction (IUGR), and sudden infant death syndrome (SIDS) among many others, the prevalence of smoking women exceeds 10% in a majority of developed countries (Dessì et al., 2018). For example, in the USA, the prevalence is about 14% (Dessì et al., 2018), and in Poland, this rate is 15–20% (Chełchowska et al., 2018). Pregnancy smoking rates can be much higher in certain sub-populations of these countries, such as for instance, among the indigenous NZ Māori population at rates of 34%—well above the national level of 10% (Humphrey et al., 2016). Smoking cessation is challenging due to multiple reasons, including nicotine addiction, cultural and social-economic status, and resistance to life-style change (Dessì et al., 2018). Early interventions are required to reverse the pathological course exposing the fetus to hazards from smoking. This demands a precise understanding of the underlying etiologies and evaluating appropriate pharmaceutical targets. We are particularly interested in pathologies emerging in the fetal cardiovascular system (CVS) due to smoking exposure (Dikalov et al., 2019). Indeed, active and passive maternal smoking cause hemodynamic changes in major fetal blood vessels such as the umbilical arteries (UAs)—a biomarker for impaired feto-placental circulation (Westergaard et al., 2001).

Unfortunately, examining the molecular and hemodynamic mechanisms of fetuses *in utero* is difficult due to ethical and technical reasons. Instead, *ex vivo* and *in vitro* experiments are performed; e.g., investigating the endothelial nitric oxide synthase (eNOS) activity in response to maternal smoking, whereby endothelial cells (ECs) were harvested from fetal umbilical veins immediately after postpartum (Andersen et al., 2004; Hedengran et al., 2018). However, *in vitro* experiments are costly, and only a limited set of experimental parameters are financially feasible. By contrast, *in silico* modeling may yield useful insights into the key aspects of CVS risks still awaiting full characterization. *In silico* models include mathematical representations of hemodynamic changes, the mechanistic stimuli and transduction pathways in the CVS, the absorption and clearance of drugs, to name a few (Chen and Popel, 2006; Barrett et al., 2012). Furthermore, some seminal discoveries of CVS diseases were made possible due to substantial method developments in DNA sequencing and corresponding bioinformatics (Joubert et al., 2016). However, applications of *in silico* models to nicotine effects on fetuses are seriously lacking. We thus here sketch a roadmap for application of multiscale *in silico* models, suggesting how the power of mathematical and computational modeling methods may augment and illuminate this significant public health problem.

## Doppler Ultrasound and Clinical Observations

Exposure to active smoking affects the systole/diastole (S/D) flow velocity ratio (S/D) in the fetal UA and the middle cerebral artery (MCA), as revealed from Doppler sonography ultrasound examinations (Albuquerque et al., 2004; Dicke et al., 2009; Milnerowicz-Nabzdyk and Bizon, 2015; Stampalija et al., 2017). In general, the S/D ratio in the UA and MCA is greater in smokers than in non-smokers, signifying a lower or absent end-diastolic flow. High S/D ratios are typical in fetuses with IUGR (Albuquerque et al., 2004). Hence, umbilical S/D ratio is used as a marker for adverse outcomes and placental abnormalities (Dicke et al., 2009). In addition, the S/D and PI (pulsatility index) in UAs are similarly reliable in fetuses >27 weeks gestation (Dicke et al., 2009).

Clinical ultrasound studies also show structural changes in the umbilical cord in pregnancies complicated by IUGR: the size of the Wharton's jelly is increased (101.60 ± 37.75 in the smoking IUGR group vs. 84.97 ± 54.16 in the control group), yet the diameters of umbilical vessels are reduced (Milnerowicz-Nabzdyk and Bizon, 2015).

## *In vitro* Techniques for Studying Nicotine Effects

### Endothelium-Dependent Vessel Relaxation

Endothelium-dependent vessel relaxation is mediated by nitric oxide (NO), which is synthesized from the amino acid L-arginine by eNOS (Chełchowska et al., 2018). The activity and concentration of the calcium-dependent eNOS were significantly lower in the umbilical vein ECs of smokers compared with non-smokers (Andersen et al., 2004). In addition, the NO and eNOS concentration in the serum of smokers were significantly lower than the non-smoking group. In contrast, inducible NOS and oxidative stress index values were higher in the smokers' group (Chełchowska et al., 2018).

### Stem Cell Technology

The rapid development of stem cell technology brings new opportunities for nicotine mechanism studies to facilitate precise treatment (Takahashi and Yamanaka, 2006). In particular, the human induced pluripotent stem cells (hiPSCs) can be derived from patient- and ethnic-specific background and hence can reproduce different kinds of cell types of human organs. For example, the change in Ca^2+^ homeostasis due to nicotine exposure was recently demonstrated by using human embryonic stem cells (hESCs; Guo et al., 2019). The same group reported that e-cigarettes caused dysfunction of hiPSC-derived ECs (Lee et al., 2019). With next generation sequencing technologies, stem cell scientists may be able to predict nicotine-induced toxicity in human cells. Moreover, ethnic-specific studies are valuable for novel mechanism discovery in those sub-populations prone to smoking exposure.

### DNA Sequencing

Development of CVS diseases is associated with up-regulation of miR-206 in smokers compared to non-smokers (Vrijens et al., 2015). *In utero* exposure to maternal smoking is also associated with DNA methylation changes and reduced neuronal development using DNA Methylation and Gene Expression Arrays (Chatterton et al., 2017). Dysregulation of key pathways critical to development is identified by using Methylation Arrays (Joubert et al., 2016). These seminal discoveries could not have been made possible without advances in bioinformatics methods.

## *In silico* Models for the CVS

### Hemodynamics Models

Numerous hemodynamics models have been developed targeting at different spatial scales of the CVS, ranging from a large vasculature with thousands of vessels (Muller et al., 2017), to a single vessel but with complex geometry such as a knotted UA (Wilke et al., 2018), or boundary conditions (van de Vosse and Stergiopulos, 2011). If the investigation focuses on the 3D wall shear stress (WSS) acting on blood vessels, then a 3D flow model may serve the purpose (Saw et al., 2017; Wilke et al., 2018). Investigating the blood flow in a vasculature proves more computationally feasible with 1D Navier-Stokes equations coupled with a wall constitutive equation (Formaggia et al., 2006; van de Vosse and Stergiopulos, 2011). To our knowledge, this method has not been applied to the fetal CVS yet, but a 3D-lumped parameter model coupled method has been used to simulate the blood flow in the ductus venosus (Leinan et al., 2013). To study pressure wave propagation along the elastic arterial wall, the flow equations are solved from the frequency domain, using methods such as the Transmission Line Theory (Muller et al., 2017), or Fourier series analysis for the velocity waveforms (Sled et al., 2019).

### Calcium Signaling Models

*In silico* models have been proposed for the effects of WSS on ion channel flux in ECs (Comerford et al., 2008; Ho et al., 2011), and on the eNOS activation and subsequent NO production (Chen and Popel, 2006). Shear stress initiates a release of intracellular ATP leading to a cascade of events including calcium (Ca^2+^) influx through intracellular store-operated Ca^2+^ channels that are key to activating NO-producing eNOS (Andrews et al., 2014). These events occur in the ubiquitous Ca^2+^ micro-domains formed in sub-plasma membrane regions known as caveolae (Cohen et al., 2004) and are the focus of multiple modeling efforts in disparate cell types (Berridge, 2006). However, while Ca^2+^ mediation of eNOS activation and production of NOS are a ripe target for mathematical modeling, we are not aware of such a model when associated with nicotine effects as mentioned above in smokers and non-smokers. Its successful application would provide important insights into the complex mechanisms described above in the fetal CVS.

### Pharmacokinetic (PK) Models

Pharmaceutical treatment of the fetal CVS requires drugs to cross the placenta barrier, then be metabolized and up-taken by target tissues. However, the fetal liver is quite limited in metabolic functions: the metabolism enzymes are simply not well-developed (Jiang et al., 2013). *In vivo* PK profile data of drugs, such as the vasodilators for treating IUGR (Sharp and Alfirevic, 2015), are very rare. Current clinical practice is by analyzing blood samples extracted from the umbilical cord but lacking any data from other organs (Huang et al., 2017). Thus, pregnant PK models which contain the maternal-placental-fetal unit can only be validated partially, i.e., in the blood compartment (Ke et al., 2012; Sharma et al., 2018). Nevertheless, predictions made from pregnant PK models for drug distribution, metabolism and clearance in fetuses are still valuable due to the use of nicotine replacement therapy in pregnant women (Benowitz and Dempsey, 2004). Notably, an *in silico* PK approach may be the only feasible option to estimate the nicotine and cotinine concentration in fetuses for ethical concerns. Nevertheless, the qualitative data of enzyme maturation (Ke et al., 2012) and the placenta transport kinetics (Prouillac and Lecoeur, 2010) are essential for any *in silico* PK model validation.

### Bioinformatics

Genomic data along with clinical correlates are being increasingly used in clinical practices as therapeutic, prognostic and diagnostic biomarkers of numerous pathologies. A meta-data analysis across 13 cohorts (*n* = 6,685) found over 6,000 of total 450,000 CpG sites were differentially methylated in relation to maternal smoking at genome-wide statistical significance (Joubert et al., 2016). Robust linear regression was used in R to evaluate the association between maternal smoking during pregnancy and cord blood DNA methylation. Covariate-adjusted statistical models are run for each cohort. Specifically, inverse variance-weighted fixed-effects meta-analysis was performed with a bioinformatics tool Metal (Willer et al., 2010).

## Discussion

In above sections, we have provided a mini-review of the current knowledge on the effects of maternal smoking on the CVS of fetuses. Although the presented data are fragmentary, they represent what we have learned from clinics (e.g., the velocity waveforms of UAs), from laboratories via tissue or blood sample analysis, and also from stem cell or DNA sequencing studies. *In silico* models, therefore, must necessarily be of a very diverse nature in order to integrate and interpret these data.

**Figure 1:**
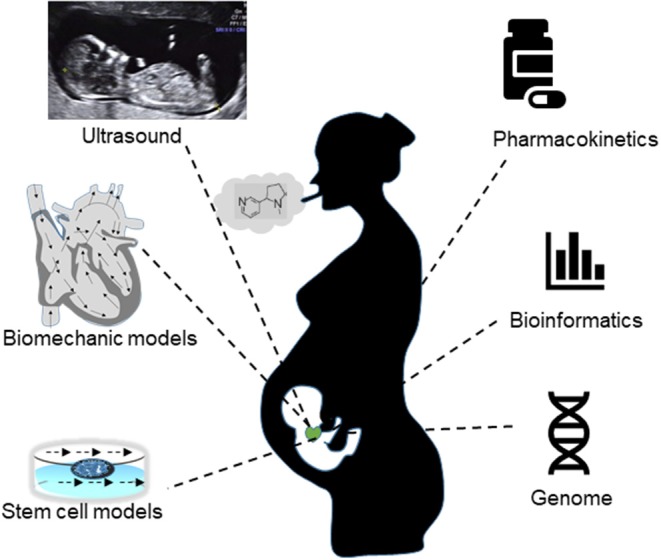
A synergy of *in vivo* measurements (ultrasound flow indices, drug concentrations), *in vitro* experiments (stem cell, DNA sequencing) and *in silico* methods (biomechanics models, pharmacokinetic models, informatics, etc.) are required to gain a better understanding of the CVS risks in fetuses exposed to active or passive maternal smoking.

Hence, in proposing a roadmap for incorporating such different datasets and models for this challenging and complex topic, we suggest at first tackling the underlying biology; particularly analyzing bio-markers for fetal CVS risks. These risk factors due to maternal smoking are confounded as well by fetal sex, race, mother age, pregnancy stage, and family disease history, to name a few. Building on this understanding, we then assemble a validated mathematical and computational model for linkage of existing experiment data, and interrogate the model to reveal novel connections between underlying mechanisms (Figure 1). More specifically, since the absent end-diastolic flows and its associated S/D ratio are viewed as the a bio-marker for adverse birth outcomes (Dicke et al., 2009), simulations with an *in silico* model may be made for normal and abnormal blood flow patterns, similar to that performed for UAs (Wilke et al., 2018), and for ductus venosus (Leinan et al., 2013) but also incorporating nicotine effect data (Dicke et al., 2009). Moreover, it is essential to connect the disparate scales of WSS and signaling dynamics in an overarching model to explore how nicotine may affect vascular wall remodeling in fetuses (Milnerowicz-Nabzdyk and Bizon, 2015).

Such a modeling framework would benefit from the philosophy of systems biology, where the analysis of physiological phenomena need to integrate the data and models from multiple spatial scales, i.e., from the molecular level to cell and organ levels (Hunter and Borg, 2003), and from bioinformatic analysis of genomic data (Joubert et al., 2016). For example, while the model for NO production and calcium signaling has to work from the protein level (Chen and Popel, 2006), a hemodynamic model for flow waveform would have to incorporate flow data in blood vessels at the organ level. To estimate the PK profile in response to nicotine, an organism level model is required for quantifying the mass exchanges between multiple compartments such as the blood, the fetus, and the placenta.

In summary, a synergy of *in vivo* clinical observations, *in vitro* experiments and *in silico* models is proposed here for revealing the underlying mechanistic mechanisms of fetal CVS risks due to maternal smoking. Collaborations from multiple research groups, and from multiple disciplines are, naturally, essential. The roadmap we sketched in this opinion paper points where the efforts—challenging as they will be—should be directed in order to tackle a health issue of such profound importance.

## Author Contributions

HH, HG, and SM drafted the manuscript. JT and PH attended the conceptual discussion of the paper and reviewed the paper.

### Conflict of Interest

The authors declare that the research was conducted in the absence of any commercial or financial relationships that could be construed as a potential conflict of interest.
